# Deep Learning Assisted Neonatal Cry Classification *via* Support Vector Machine Models

**DOI:** 10.3389/fpubh.2021.670352

**Published:** 2021-06-10

**Authors:** Ashwini K, P. M. Durai Raj Vincent, Kathiravan Srinivasan, Chuan-Yu Chang

**Affiliations:** ^1^School of Information Technology and Engineering, Vellore Institute of Technology (VIT), Vellore, India; ^2^Department of Computer Science and Information Engineering, National Yunlin University of Science and Technology, Yunlin, Taiwan

**Keywords:** convolutional neural network, infant cry classification, short time fourier transform, support vector machine, spectrogram

## Abstract

Neonatal infants communicate with us through cries. The infant cry signals have distinct patterns depending on the purpose of the cries. Preprocessing, feature extraction, and feature selection need expert attention and take much effort in audio signals in recent days. In deep learning techniques, it automatically extracts and selects the most important features. For this, it requires an enormous amount of data for effective classification. This work mainly discriminates the neonatal cries into pain, hunger, and sleepiness. The neonatal cry auditory signals are transformed into a spectrogram image by utilizing the short-time Fourier transform (STFT) technique. The deep convolutional neural network (DCNN) technique takes the spectrogram images for input. The features are obtained from the convolutional neural network and are passed to the support vector machine (SVM) classifier. Machine learning technique classifies neonatal cries. This work combines the advantages of machine learning and deep learning techniques to get the best results even with a moderate number of data samples. The experimental result shows that CNN-based feature extraction and SVM classifier provides promising results. While comparing the SVM-based kernel techniques, namely radial basis function (RBF), linear and polynomial, it is found that SVM-RBF provides the highest accuracy of kernel-based infant cry classification system provides 88.89% accuracy.

## Introduction

Babies convey their needs through cries. Experienced baby care persons and parents can understand the reason for the baby's cries. Some young working parents struggled to interpret the baby's cries. The baby's cries imply their emotions, physical needs, and pathological problems from internal or external stimulation. Humans can listen to the audio signal in the frequency range from 50 to 15,000 Hz for music, 20 to 20,000 Hz for sounds, and 100 to 4,500 Hz for speech. Within this range, humans can discriminate the audio. Babies do not have control over their vocal tract so that it is more sensitive than adults. Baby cries contain information, and their crying pattern varies based on their physical and emotional state. The researchers found that there is a pattern for each kind of cry. Infant cry classification can be considered pattern recognition or speech recognition. An abnormal cry of the infant can indicate a genetic or pathological problem. Childcare experts can differentiate it. The baby cry-based recognition approach will help us know the infant's feelings from their cries. Techniques such as signal preprocessing, feature extraction, feature selection, and classification are the steps involved in baby cry classification.

Signal preprocessing is crucial to eliminate the unwanted signal present in the audio signals. The audio signal features can be analyzed based on their time, frequency, and time-frequency domain. Neural networks can be able to learn features from the audio itself. The spectral representation of audio plays a crucial role in the classification of audio signals using neural networks. The initial work of infant cry classification was started in the 1960s. Kia et al. (1) designed a system to detect a baby's cries using fast Fourier transform with a fuzzy classifier. Petroni et al. (2) attempted to distinguish the baby's anger, fear, and pain cries. The features were extracted from the Mel cepstrum coefficient, and four kinds of neural networks such as time-delay network, feed-forward network, cascade network, and recurrent network were implemented; the results showed that a fully connected neural network gave a better performance.

Mima and Arakawa (3) examined the frequency analysis of infant cries (hunger, discomfort, sleepiness) and found the difference in Fourier transform tendencies for each state. Jam and Sadjedi (4) carried out work to distinguish the pain and normal infant cries. They observed that, while processing the audio signal, silence elimination, filtering, pre-emphasizing was crucial. Mel frequency and entropy based on multibands were used in the extraction of features. Principal component analysis reduces the dimensions of the feature vector. Multilayer perceptron recognized the infant cries and achieved better results because multiband entropy provides entropy distribution in the spectrum. The work was focused on the linear and the non-linear feature coefficient technique to detect and classify the normal and hearing impaired infant cries. The linear feature coefficients were extracted from linear predictive coefficient (LPC), and those features were optimized by using the hereditary approach. The bilinear nilpotent technique was used to analyze the non-linear signal. Kernel discriminant analysis (KDA) transforms those features into a low-dimensional basis to show the linear and non-linear features' contribution. Support vector machine (SVM) and expectation-maximization (EM) algorithms over an expert system were employed to classify the data. It shows that non-linear feature with an expert system-based classification approach gives better performance (5).

In previous works, the audio signal involves numerous preprocessing techniques; feature extraction and feature selection techniques were used to classify the data. However, deep learning approaches automatically extract the raw data features, even without additional preprocessing methods. Implementing the deep learning approach needs millions of data samples to get the best results. Moreover, this motivates us to enhance the infant cry classification model's performance even with the small dataset by extracting the features using the deep learning technique and classifying the infant cries using a machine learning algorithm with less computational complexity. This work classifies the most common infant cries such as hunger, pain, and sleepiness.

## Related Works

It is a crucial task to discriminate the infant cries, so in this work (6), dealt with K-NN classifier with features such as short-time energy, harmonic to average power ratio (HAPR), Mel frequency coefficient, and harmonicity factor (HF) to recognize the infant cry sounds. In this work (7), convolutional restricted Boltzmann machine was used to analyze the unsupervised auditory filter banks. The network consists of the visible and hidden layers, and the weights were shared between those layers. The non-linear activation of Noisy Leaky Rectifier Linear Unit (NLReLU) was used. The parameters of the network were optimized by using the Adam optimization method. Convolutional restricted Boltzmann machine and discrete cosine transform were applied to reduce the feature dimensions. Those features were compared with MFCC features, and it was found that CNBM-based feature performs well in the discrimination of healthy and pathological auditory cries. In this case (8), they employed a convolutional neural network in infant cry vocalizations. The cry segments were manually extracted from the audio signal and segmented into a 4–8-s duration of segments. Audio signals were represented as spectrogram through short-time Fourier transform, which is based on Fourier transform. The spectrogram is the input for convolutional neural network. The convolution layer can obtain the features from the spectrogram, and the network can successfully discriminate the baby cry vocalizations.

This study (9) investigated the feature extracted from wavelet packet transform based on complex dual-tree form to discriminate the three sets of infant cries such as normal vs. asphyxia, normal vs. deaf, and hunger vs. pain. Various feature selection techniques such as correlation feature selection, principal component analysis, and information gain were applied to select the most relevant and essential features. Extreme machine learning can successfully classify infant cry patterns. This work (10) presented the combined acoustic and prosodic features to distinguish the audio signal's variations. Merge those features and generate a feature matrix for the deep neural network. MFCC features were considered to present the acoustic features. The features such as fundamental frequency, intensity, and formats carry the prosodic feature information. The neural network has an input, two hidden, and an output layer to calculate the weighted prosodic features. Those features were taken as input to the deep learning approach, which is found that the merged features can distinguish the variation present in infant cry signals.

Priscilla Dunstan found that every baby makes certain sounds while crying to convey their needs, such as Owh Heh, Eh, Eair, and Neh, representing tired, discomfort, burp or sleepy, pain, and hunger. Dewi et al. (11) analyze the feature extraction techniques such as linear frequency cepstral coefficient and Mel frequency cepstral coefficient. It extracts the features from the spectrogram. Vector quantization, KNN, and neural network were deployed in the classification of infant cries. It is found that LFCC with KNN classifier gives a better result than other techniques. Felipe et al. (12) discussed the motivation concerning the classification of infant cries. The local visual features such as binary pattern, robust binary pattern, phase quantization, Mel frequency cepstrum coefficient, Mel scale features, and constant Q chromogram were considered to obtain the features from the spectrogram. The best result was obtained from the local binary pattern using SVM with an accuracy of 71.68%. Gujral et al. (13) analyzed and fine tuned the neural network using the transfer learning approach to recognize infant cries. Long short-time memory (LSTM) and convolutional neural network (CNN) were analyzed with and without transfer learning. It is observed that transfer learning based CNN outperforms LSTM with an average recall of 75.7%. The latent factor approach can efficiently obtain the information from high-dimensional and sparse data. A multilayered and randomized latent factor model was adopted to reduce the time complexity and enhance data representation for better understanding. In the case of nonnegative data, β divergence latent factor model is adopted to analyze the performance in recommender systems (14–17).

## Materials for Feature Extraction and Classification

**Figure 1:**
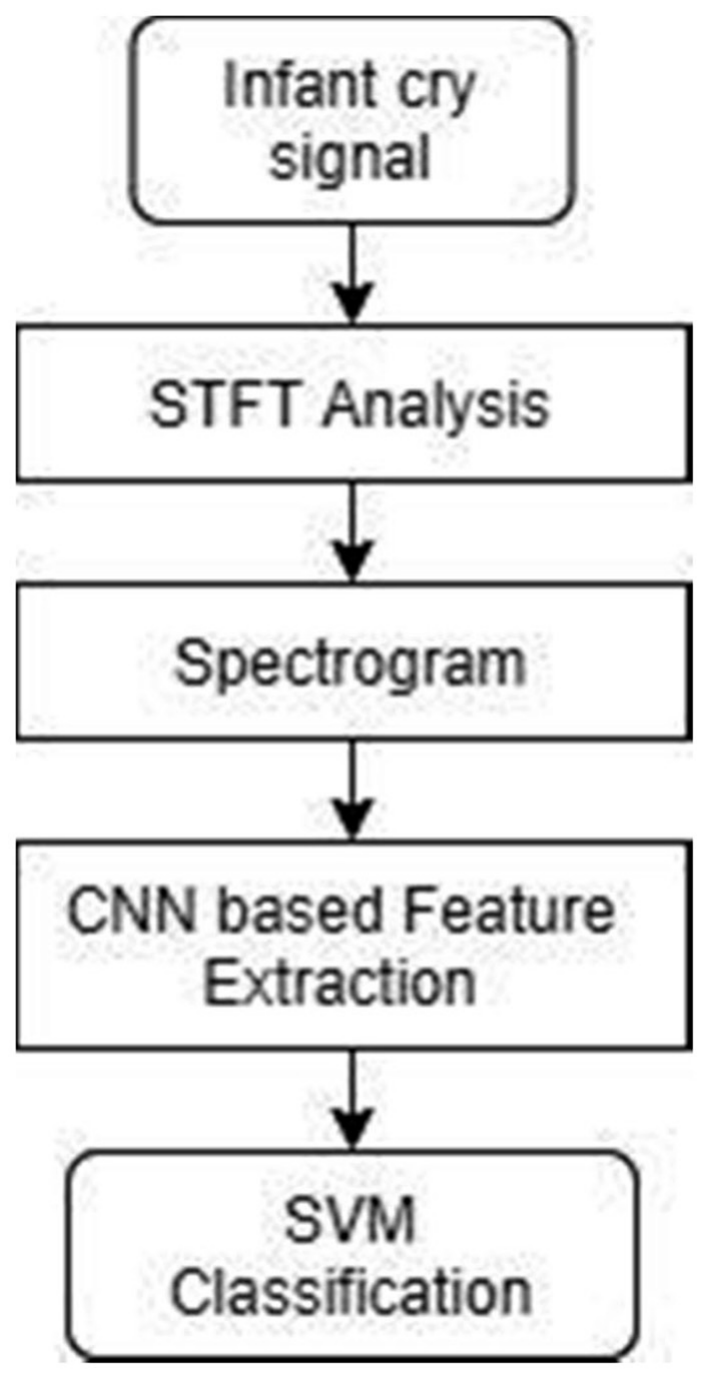
Work flow diagram for baby cry classification.

In our approach, the infant cry signals are taken as input, and short-time Fourier transform (STFT) is deployed to convert the neonatal cry signals into the spectrogram image. The features are extracted from the image using a deep convolutional neural network. Furthermore, the SVM classifier discriminates the neonatal cries as pain, hunger, and sleepiness. Figure 1 represents the procedure involved in the infant cry classification system.

### Audio Signal Analysis

**Figure 2:**
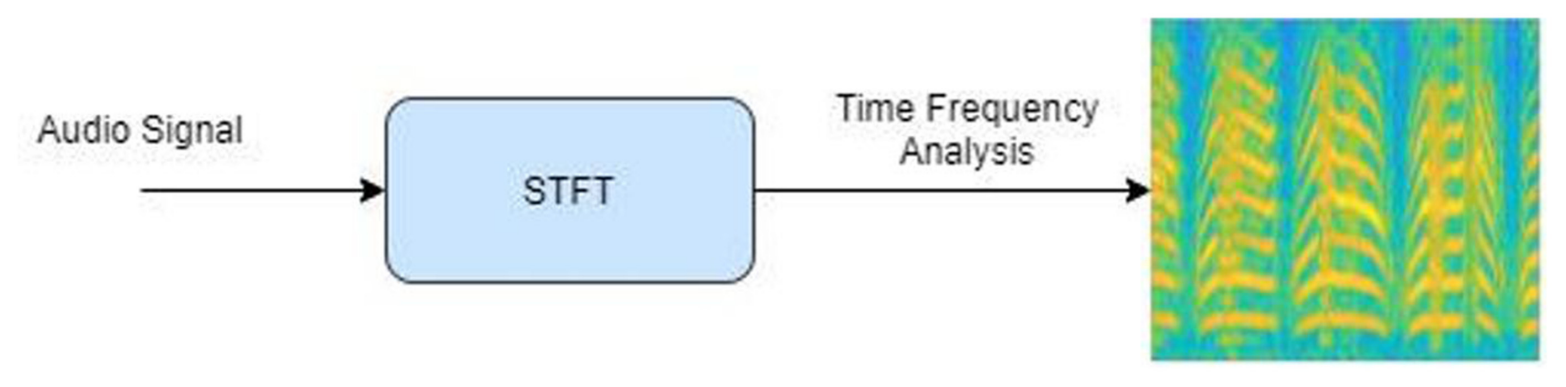
Audio signal transform into spectrogram.

The frequency content of the audio signal varies by time. For that, a standard technique is required to analyze the signal in time-frequency domain. Fourier transform is deployed to characterize the time-varying frequency content present in the signal. It analyzes the signal by converting the domain of time to frequency. As a result of applying fast Fourier transform (FFT), the signal phase and magnitude are obtained. The FFT length of the signal must be equal to two times the power of length needed to get a good frequency resolution in FFT. The STFT simultaneously examines the time and frequency content of the signal. The signals are breakdown into numerous segments called frames; then, each segment multiplied with a window either with or without overlapping. Spectral leakage will happen while converting the signal from time into a frequency domain. The windowing function tries to reduce the spectral leakage and unusual discontinuity in the signal due to segmentation. Several windowing functions are there, such as Hamming, Blackman, uniform, flattop, and exponential. STFT computes the Fourier transform for each windowing segment. The magnitude square of the STFT is spectrogram. It represents the distribution of frequencies present in the signal changes over time (18–21). Short-time Fourier transform and spectrogram are mathematically described as follows.

$$\begin{array}{l} F\left(m,\omega \right)=\sum x\left(n\right)w{\left(n-m\right)}^{e^{-j\omega n\;}}\\ S\left(m,\omega \right)=|F\left(m,\omega \right){|}^{2} \end{array}$$

whereas, *F*(*m*, ω) is the short-time Fourier transform, *x*(*n*) represents a signal, *w*(*n*–*m*) represents the windowing technique, and *S*(*m*, ω) is the spectrogram. Figure 2 illustrates the process involved in audio signal analysis.

### Convolutional Neural Network

The classical neural network comprises input, hidden, and output layers (22). The data has passed from one layer to another layer. Every layer has several nodes; each node takes a set of values from previous as input and does some mathematical operation that produces a single value as an output to the consecutive layer's nodes. In convolutional network, the image itself is taken as an input and generates an image as an output. It breaks the image into features and detects the particular pattern from that image.

**Figure 3:**
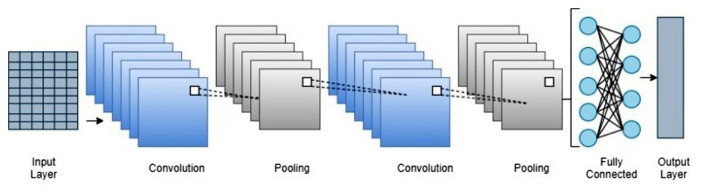
Pictorial representation of convolutional neural network.

Furthermore, it comprises convolution, pooling, and a fully connected layer. The convolution layer performs convolution with the help of filters. Each node has its own filters, and it extracts the features from the image. It is succeeded by a non-linear function (RELU, sigmoid, tanh) that performs threshold operation. Pooling tends to minimize network complexity. Max pooling and average pooling are types of pooling. It gathers the part of images into small rectangular portions and examines the highest values in max pooling. In average pooling, it calculates the median value of that specific rectangular portion. The rectangular regions are the kernels. The fully connected layer carries information about the number of output classes, and it maps the data to its output. The softmax layer normalizes the data and gives the score a probability for that input to every output data. The classification layer produces a result based on the probability score (23, 24). Figure 3 shows the simple convolutional neural network.

### Support Vector Machine

**Figure 4:**
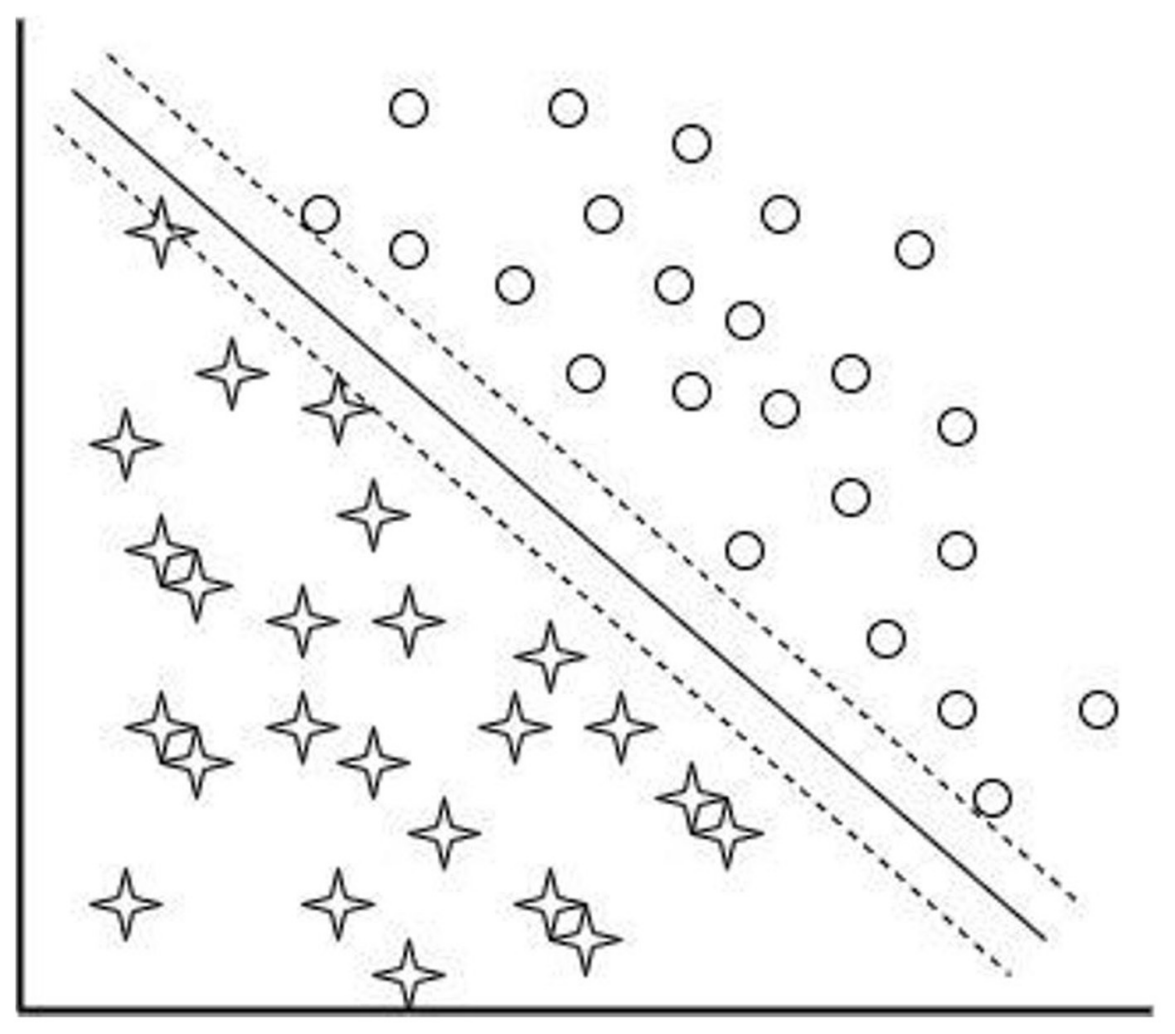
Pictorial representation of linear SVM.

SVM executes classification by differentiating the data points with a larger margin using hyperplane as a decision boundary. SVM classifier includes hyperplane, margin hyperplane, kernels, and soft margin. The hyperplane is the line that differentiates the discrete data points. Margin is the distance between data samples and the hyperplane. The margin hyperplane divides the dissimilar data with maximum distance from one another. The data samples which are near the hyperplane are named as support vectors. Soft margin in SVM creates accurate models from data that are not able to generalize. The purpose of kernel function converts the data samples into high dimensional feature space. Linear SVM and non-linear SVM are the categories involved in SVM. Figure 4 shows the simple linear SVM model. In linear SVM, the data points can be distinguishable with a simple straight line. It can be defined as

$$\begin{array}{l} w\cdot a+b=0 \end{array}$$

where, “*w*” represents the adjustable weight, “*a*” defines the input data, and “b” indicates the bias.

When the data samples are not separable using a straight line, non-linear SVM is used to solve the non-linear problems. For that, the kernel functions are used to modify the data into higher feature space. The most common kernel functions are linear, quadratic, polynomial, and radial basis function (RBF) kernel. Linear kernel combines all support vectors linearly to produce the output. It can be described as

$$\begin{array}{l} G\left(x_{i},x_{j}\right)={x_{i}}^{\prime }xj\; \end{array}$$

The quadratic kernel does not require any changes in the parameter to get an efficient result. The polynomial kernel considers each support vector and computes its kernel function. In the polynomial kernel, the polynomial order is usually chosen by more than one. If the polynomial order is one, then it will become a linear kernel. It is mathematically defined as

$$\begin{array}{l} G\left(x_{i},x_{j}\right)={\left({x_{i}}^{\prime }x_{j}+1\right)}^{p} \end{array}$$

**Figure 5:**
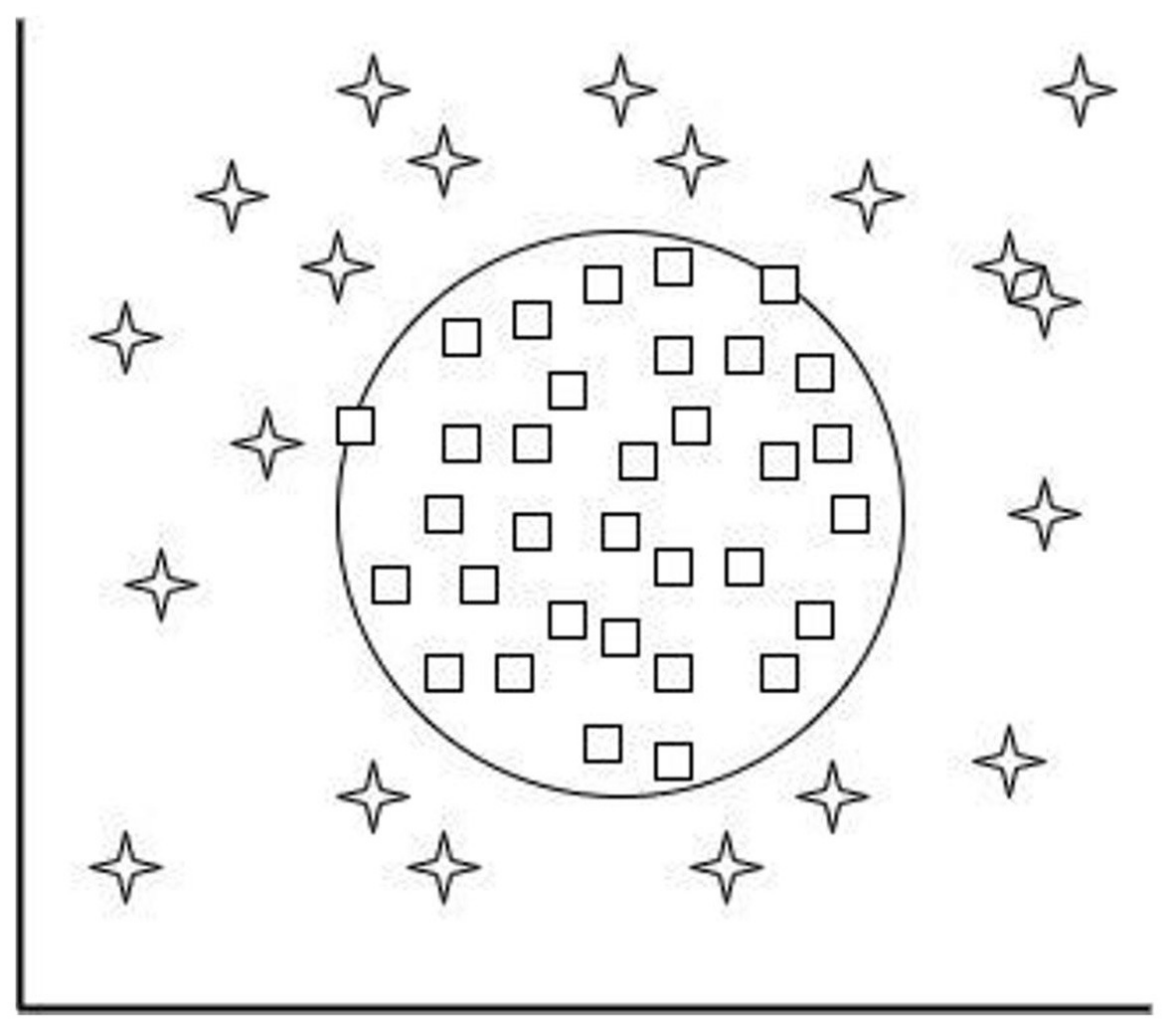
Pictorial representation of non-linear SVM.

RBF kernel can effectively generalize the data and perform better to solve the practical problem. In the RBF kernel, the support vectors can automatically determine the number of RBF and its centers. It can be represented as

$$\begin{array}{l} G\left(x_{i},x_{j}\right)=\exp\left(-\|x_{i}-x_{j}{\|}^{2}\right) \end{array}$$

where, *x*_*i*_ and *x*_*j*_ represent the observations and *p* represents the polynomial order. SVM optimization can be performed by increasing the margin space between data samples and selecting the precise kernel function for our system demands (25, 26). Figure 5 shows the non-linear SVM model.

## Results and Discussion

**Figure 6:**
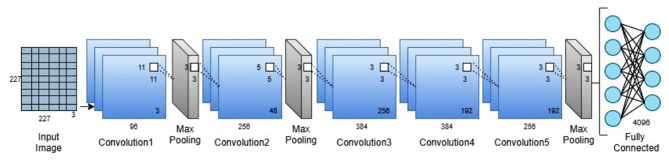
Convolutional neural network for feature extraction.

The dataset of pain, hunger, and sleepiness cries was collected from the infants born in National Taiwan University Hospital Yunlin Branch, Taiwan (27, 28). The infants' cries were recorded from the healthy infants' age range from 1 to 10 days. There were no pathological problems or any complications found in the babies, even during birth and after birth. In this study, we consider 300 audio records, in that every 100 audio samples for hunger, pain, and sleepiness cries. Each audio signal is in the length of 4 s data with a sampling frequency of 8 kHz. Eighty percent of the data is utilized for training, and the remaining data is used for testing. The whole experiment is implemented in MATLAB using the Deep Learning Toolbox and Statistics and Machine Learning Toolbox. The convolutional neural network-based feature extraction process is shown in Figure 6.

**Figure 7:**
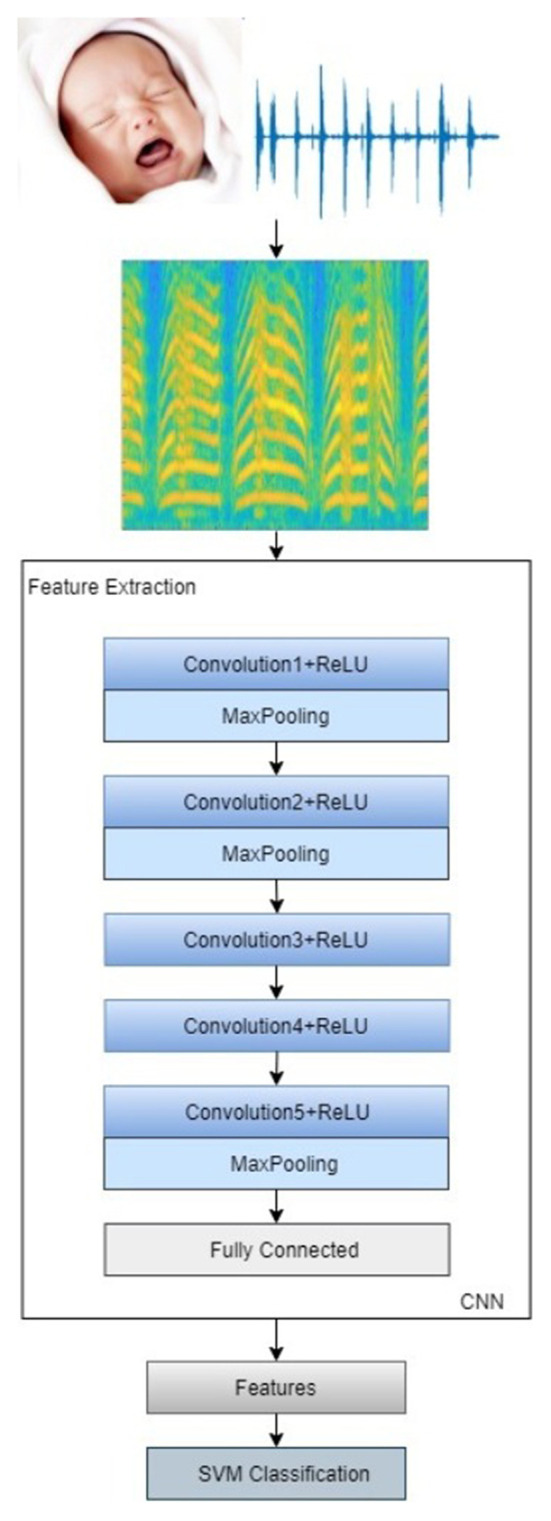
Proposed infant cry classification system.

At first, the neonatal cry auditory signals are transformed into spectrogram images by applying the short-time Fourier transform. The audio signals are broken down into numerous segments called frames; then, the windowing function multiplies with each frame. In this study, the auditory signals are segmented into 128 sections with 64 windows overlapping. The Hamming window function is deployed here. Fourier transform is computed; for that purpose, 256 discrete Fourier transform points are considered. The magnitude square of the STFT gives the spectrogram image. Figure 7 illustrates the overall process involved in this study.

The data augmented is done to resize the image into 227^*^227^*^3 to meet the pretrained deep convolutional network's requirement. The convolutional neural network comprises eight layers, five convolution layers succeeded by RELU activation layer and pooling, and three fully connected layers. The deep network breaks the images into features using multiple sets of layers. The foremost layer of the network is taking the input data and normalizes the input image. The first layer of convolution has 96 filters; each filter size is 11^*^11 with four strides and zero padding, succeeded by RELU activation and max pooling of size 3^*^3 with a stride of two. The second layer of convolution comprises 256 filters, 5^*^5 filter size with one stride, succeeded by RELU and max pooling, in which 3^*^3 pooling size with two strides and zero paddings. The third layer of convolution has 384 filters, 3^*^3 filter size with one stride and one padding succeeded by RELU. The fourth layer of convolution consists of 384 filters, 3^*^3 size of filters with one stride succeeded by the RELU layer. The last layer of convolution has 256 filters, 3^*^3 size of filters with one stride succeeded by RELU and max pooling, in which 3^*^3 pooling size with two stride and zero paddings. The convolution layer description is shown in Table 1. We get the features from a fully connected layer instead of convolution, making it easier to execute the model with crucial features. The obtained features from the convolutional network are fed into the machine learning classifier. SVM with several kernel techniques such as polynomial, linear, and radial basis function is implemented to discriminate the baby cries. We have used error correcting output code (ECOC) approach with a one vs. one coding design to train the multiclass SVM model. In this case, we have considered three kinds of baby cries, for that the approach yields three binary learners which use all combinations of infant cries and return a multiclass model. To avoid overfitting or underfitting, we had cross validated the model using 10-fold cross validation, efficiently estimating the model with conventional variance.

**Table 1:** Convolution layer description of the network.

**Layer number**	**Layers**	**Number of filters**	**Filter size**	**Number of channels**
1	Conv1	96	11*11	3
2	Conv2	256	5*5	48
3	Conv3	384	3*3	256
4	Conv4	384	3*3	192
5	Conv5	256	3*3	192

The confusion matrix analyzes the performance of the approach. It has attributes such as true positive (TP), false positive (FP), false negative (FN), and true negative (TN). The 3^*^3 confusion matrix is defined in Table 2 where, TP_A_ defines the number of samples that are classified as class A. TP_B_ represents the number of data samples that are correctly recognized in class B. TP_C_ describes the number of data samples that are precisely classified in class C. *E*_AB_ represents the number of data samples from class A which are mislabeled as class B. *E*_AC_ describes the number of samples from class A that are misinterpreted as class C. *E*_BA_ represents the number of data samples from class B which are mislabeled as class A. *E*_BC_ describes the number of data samples from class B that are mislabeled as class C. *E*_CA_ represents the number of data samples from class C which are misinterpreted as class A. *E*_CB_ defines the number of data samples from class C that are misinterpreted as class B. For class A, false negative (FN_A_), false positive (FP_A_), and true negative (TN_A_) can be calculated as

$$\begin{array}{l} {\text{FP}}_{\text{A}}=E_{\text{AB}}+E_{\text{AC}}\\ {\text{FN}}_{\text{A}}=E_{\text{BA}}+E_{\text{CA}}\\ {\text{TN}}_{\text{A}}=E_{\text{BC}}+E_{\text{CB}}+{\text{TP}}_{\text{B}}+{\text{TP}}_{\text{C}} \end{array}$$

**Table 2:** 3*3 confusion matrix.

	**A**	**B**	**C**
A	TP_A_	E_AB_	E_AC_
B	E_BA_	TP_B_	E_BC_
C	E_CA_	E_CB_	TP_C_

For class B, false negative (FN_B_), false positive (FP_B_), and true negative (TN_B_) can be calculated as

$$\begin{array}{l} {\text{FP}}_{\text{B}}=E_{\text{BA}}+E_{\text{BC}}\\ {\text{FN}}_{\text{B}}=E_{\text{AB}}+E_{\text{CB}}\\ {\text{TN}}_{B}=E_{\text{CA}}+E_{\text{AC}}+{\text{TP}}_{\text{A}}+{\text{TP}}_{\text{C}} \end{array}$$

For class C, false negative (FN_C_), false positive (FP_C_), and true negative (TN_C_) can be calculated as

$$\begin{array}{l} {\text{FP}}_{\text{C}}=E_{\text{CA}}+E_{\text{CB}}\\ {\text{FN}}_{\text{C}}=E_{\text{AC}}+E_{\text{BC}}\\ {\text{TN}}_{\text{C}}=E_{\text{AB}}+E_{\text{BA}}+{\text{TP}}_{\text{A}}+{\text{TP}}_{\text{B}} \end{array}$$

Those are used to compute the performance metrics such as precision, accuracy, recall, F1 score, and specificity. Accuracy compares the actual and desired output. Specificity shows the proportions of all negative cases, and recall represents the proportions of all positive cases. Precision shows the proportions of positive which are actually positive. F measure/F1 score computes the mean of recall and precision.

$$\begin{array}{l} \text{ Accuracy}=\frac{\left(\text{TN}+\text{TP}\right)}{\left(\text{TP}+\text{FP}+\text{FN}+\text{TN}\right)}\\ \text{ Recall}=\frac{\text{TP}}{\left(\text{FN}+\text{TP}\right)}\\ \text{Specificity}=\frac{\text{TN}}{\left(\text{TN}+\text{FP}\right)}\\ \text{ Precision}=\frac{\text{TP}}{\left(\text{FP}+\text{TP}\right)}\\ \text{ F}1\text{ Score}=\frac{2\text{TP}}{\left(2\text{TP}+\text{FP}+\text{FN}\right)} \end{array}$$

Tables 3–5 represent the performance metrics such as specificity, sensitivity, precision, accuracy, and F1 score of infant cries based on SVM-RBF, polynomial, and linear kernels.

**Table 3:** Performance evaluation of SVM-RBF.

**Performance metrics**	**Hunger**	**Pain**	**Sleepy**	**Average measures**
Specificity	0.8571	0.8235	1.0000	0.8935
Sensitivity	0.9032	0.9643	0.9677	0.9450
Precision	0.8000	0.9333	0.9333	0.8888
Accuracy	0.8889	0.9111	0.9778	0.9259
F1 score	0.8276	0.8750	0.9655	0.8893

**Table 4:** Performance evaluation of SVM-polynomial.

**Performance metrics**	**Hunger**	**Pain**	**Sleepy**	**Average measures**
Specificity	0.8125	0.8750	0.9231	0.8702
Sensitivity	0.9310	0.9655	0.9063	0.9342
Precision	0.8667	0.9333	0.8000	0.8666
Accuracy	0.8889	0.9333	0.9111	0.9111
F1 score	0.8387	0.9032	0.8571	0.8663

**Table 5:** Performance evaluation of SVM-linear.

**Performance metrics**	**Hunger**	**Pain**	**Sleepy**	**Average measures**
Specificity	0.8125	0.8571	0.8667	0.8454
Sensitivity	0.9310	0.9032	0.9333	0.9225
Precision	0.8667	0.8000	0.8667	0.8444
Accuracy	0.8889	0.8889	0.9111	0.8963
F1 score	0.8387	0.8276	0.8667	0.8443

**Figure 8:**
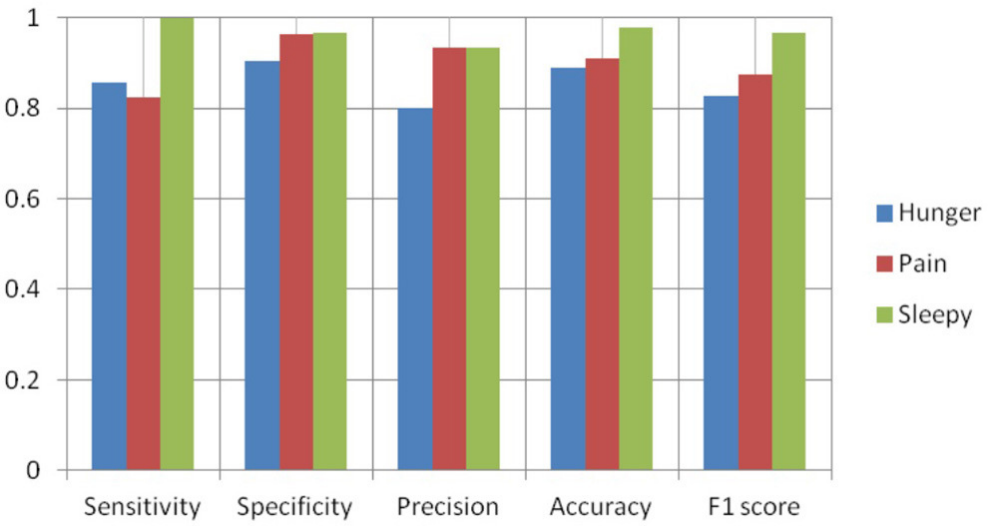
Performance measures of SVM-RBF.

**Figure 9:**
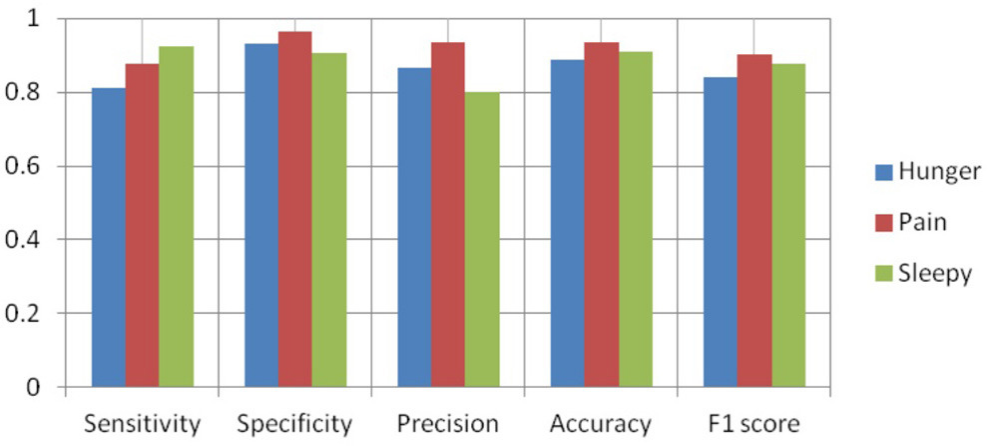
Performance measures of SVM-polynomial.

**Figure 10:**
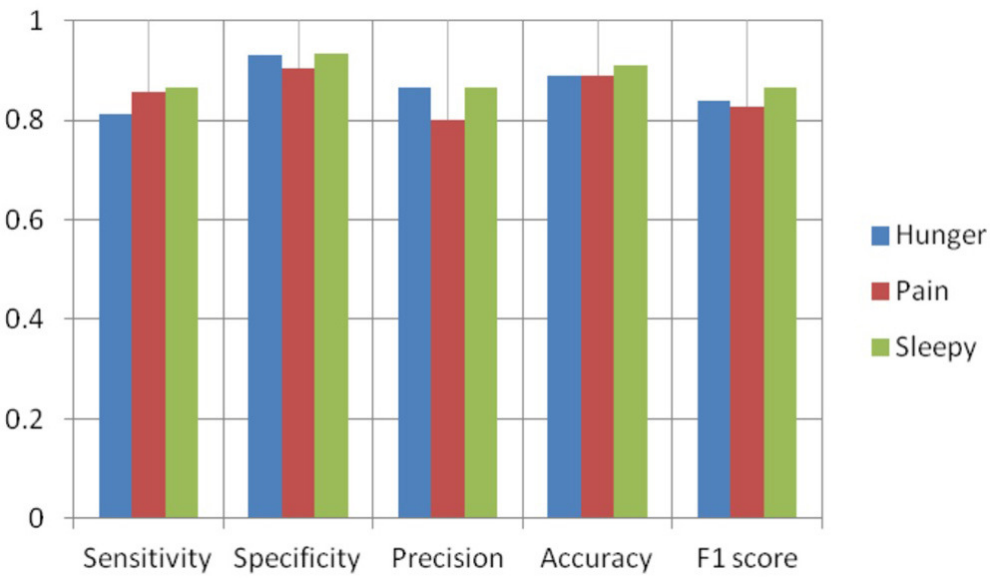
Performance measures of SVM-linear.

Figures 8–10 show the CNN-SVM-based infant cry classification model's performance measures with various kernel functions. It is clearly shown that SVM with RBF performs better than other kernel functions. Overall, the deep convolutional network-based feature extraction and SVM with the RBF classification-based model by the parameters of *c* = 1 and gamma = 1 provides the highest accuracy of 88.89% with a generalized classification error of 5.56% and standard deviation of 0.0835.

**Figure 11:**
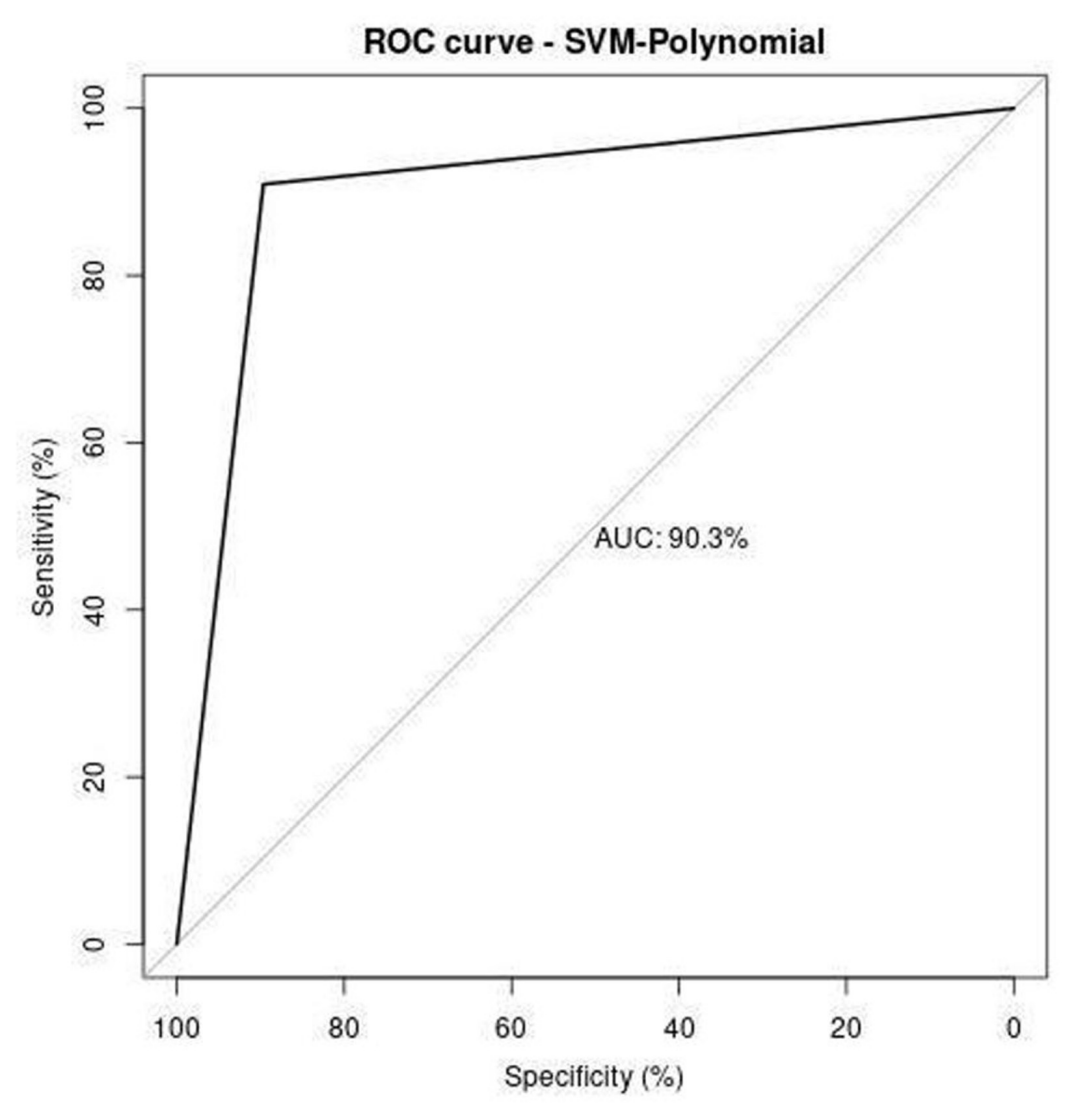
ROC analysis of SVM-polynomial.

**Figure 12:**
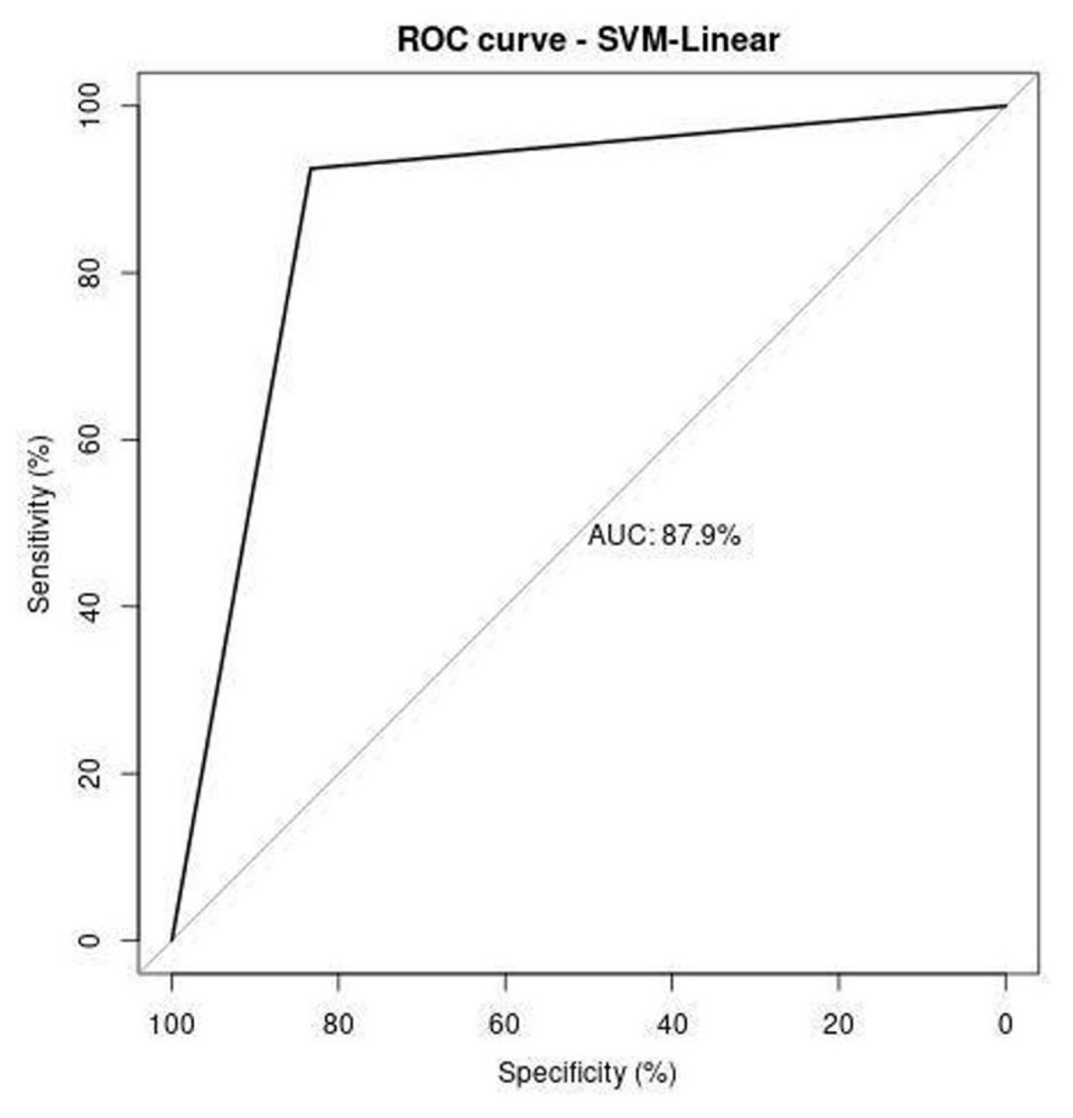
ROC analysis of SVM-linear.

**Figure 13:**
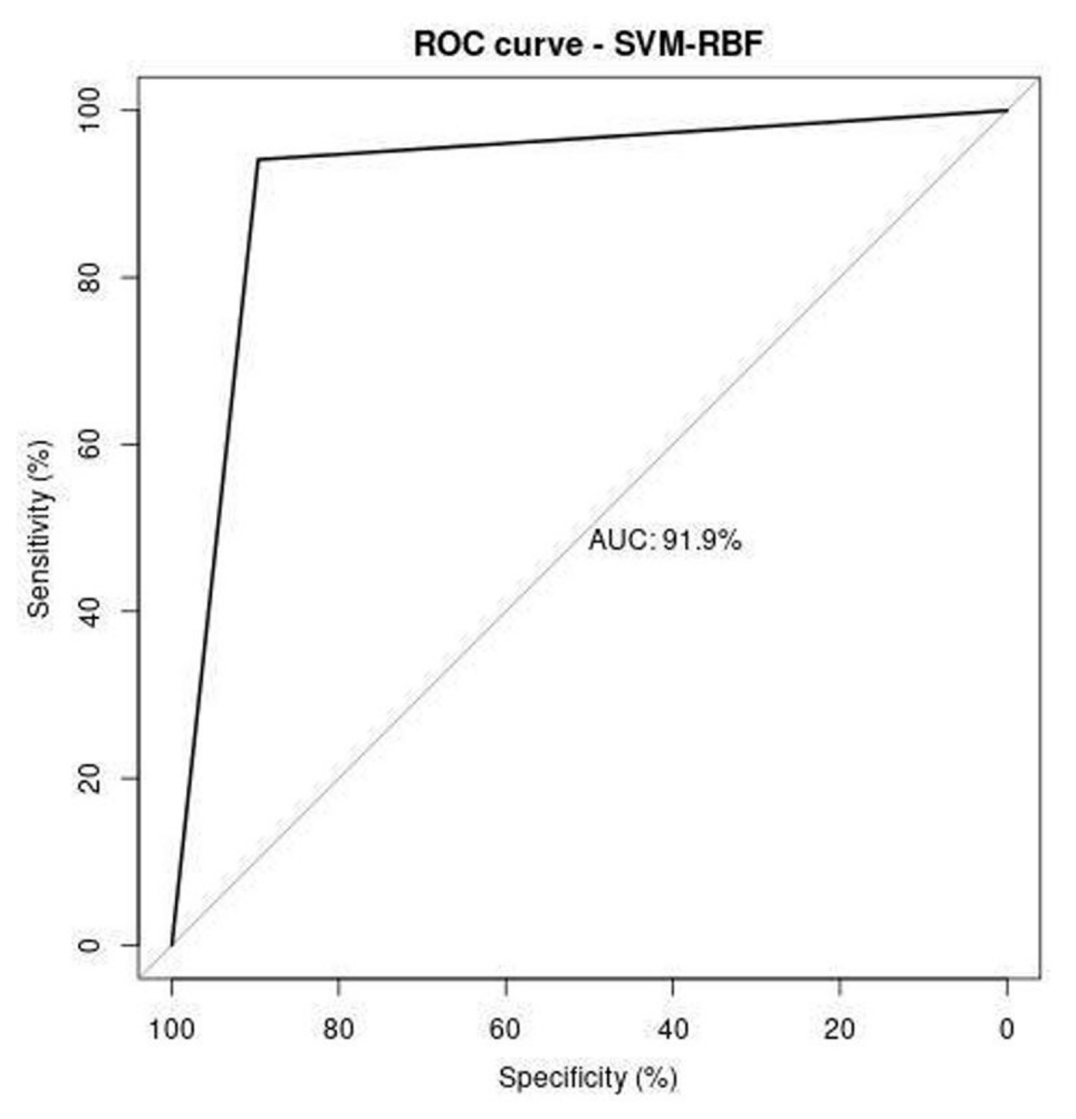
ROC analysis of SVM-RBF.

Receiver operating characteristics (ROC) curve illustrates the correlation between the true-positive rate (TPR) and false-positive rate (FPR). It is a crucial tool to estimate the performance of the approach. Figures 11–13 represent the ROC curve for SVM-based polynomial, linear, and RBF kernel. The area under the curve for the polynomial kernel is 90.3%, the linear kernel is 87.9%, and the RBF kernel is 91.9%.

**Figure 14:**
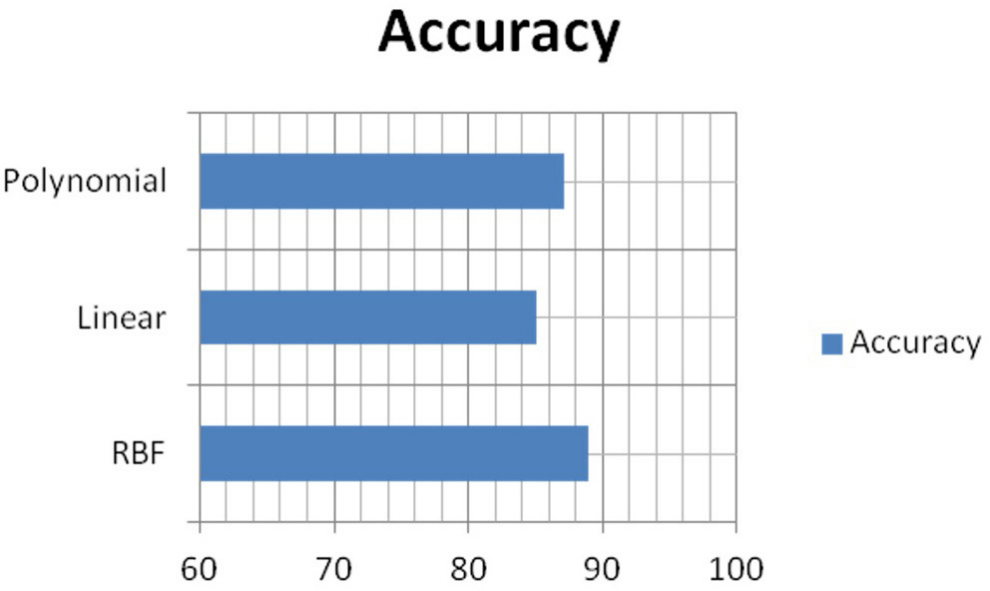
Comparison of various kernels in SVM.

Figure 14 shows the comparison of overall accuracy obtained from various kernel functions in SVM. SVM-polynomial, linear, and RBF kernel's accuracy is 86.67, 84.44, and 88.89%. It clearly shows that the performance of the SVM-RBF kernel gives more accurate results than other kernel functions. We observe that by varying the kernel functions in SVM, the classification system's performance changes drastically.

In the SVM polynomial kernel, the 3rd and 4th order of polynomial gives an accuracy of 86.67%, the 5th order acquires 84.45%, and the 6th polynomial order provides 82.22%. It is observed that the variations in the polynomial order affect the performance of the model. By increasing the polynomial order, the system's performance (accuracy) gradually decreases. In the classification of infant cries' physiological needs, the pretrained network, which uses stochastic gradient descent, acquires 76% accuracy, and stochastic gradient descent with momentum obtained 82% accuracy (29). It is also compared with convolutional neural network feature extraction based on other machine learning techniques such as KNN, Naïve Bayes, and Decision Tree, which acquire 84.69, 83.56, and 84.45% accuracy. While comparing this CNN architecture with another pretrained CNN architecture which comprises 13 convolution layers followed by three fully connected layers, the features were extracted based on those layers. They passed the features to the SVM classifier, which gives 87.22% accuracy, respectively. MFCC was used to analyze the infant cry audio signal, which acquires an accuracy of 85.76%. It is found that STFT outperformed baseline MFCC. Compared with these, the proposed approach gives better performance than the existing approach concerning infant cry classification and the SVM classifier performs better than KNN and Naïve Bayes. It is observed that the time taken to train the pretrained network takes more time than the convolutional feature extraction-based machine learning classification. The neonatal cry classification model helps the new parents to know their infants once they discover the need for baby cries. They can respond to their baby's needs more quickly and effectively.

## Conclusion

Infant cries carry information about the infant's feelings. This study combines the deep learning and machine learning model to enhance the infant cry classification model's efficiency even with small datasets. The audio cry signals are converted into a spectrogram image using STFT. The spectrogram images are fed into the deep convolutional network. The convolutional network is good at extracting features from images. The extracted features are taken as input for the SVM technique. The experimental result exhibits that the proposed method acquires the highest classification accuracy of 88.89% compared with all other approaches considered in the literature. It is found that CNN can extract the features from the time-frequency representation of audio signals. To the best of the authors' knowledge, the demonstration of CNN feature extraction and machine learning classifier is reported for the first time in infant cry classification. Convolutional feature extraction-based machine learning classifier provides good results even with the moderate dataset, but tuning the SVM technique's hyperparameters is computationally expensive. In the future, we would like to experiment with this deep neural network feature extraction with hybrid or embedded machine learning based classifiers. Also, much more focus will be given to implementing the machine learning model's optimization techniques, which may enhance these approaches' efficiency.

## Data Availability Statement

The original contributions generated for this study are included in the article/supplementary material, further inquiries can be directed to the corresponding author/s.

## Ethics Statement

The studies involving human participants were reviewed and approved by Human Research Ethics Committee at National Cheng Kung University, Taiwan. Written informed consent to participate in this study was provided by the participants' legal guardian/next of kin.

## Author Contributions

C-YC: conceptualization, resources, project administration, and funding acquisition. PV and AK: methodology and software. KS and C-YC: validation and writing—review and editing. PV: writing—original draft preparation. All authors contributed to the article and approved the submitted version.

## Conflict of Interest

The authors declare that the research was conducted in the absence of any commercial or financial relationships that could be construed as a potential conflict of interest.

## References

[1] Kia M Kia S Davoudi N Biniazan R. A detection system of infant cry using fuzzy classification including dialing alarm calls function. In: Second International Conference on the Innovative Computing Technology (INTECH 2012). Casablanca: IEEE (2012) p. 224–9. 10.1109/INTECH.2012.6457776

[2] Petroni M Malowany AS Johnston CC Stevens BJ. Classification of infant cry vocalizations using artificial neural networks (ANNs). In: International Conference on Acoustics, Speech, Signal Processing. Detroit: IEEE (1995) p. 3475–8. 10.1109/ICASSP.1995.479734

[3] Mima Y Arakawa K. Cause estimation of younger babies' cries from the frequency analyses of the voice-Classification of hunger, sleepiness, and discomfort. In: International Symposium on Intelligent Signal Processing and Communications. Yonago: IEEE (2006) p. 29–32. 10.1109/ISPACS.2006.364828

[4] Jam MM Sadjedi H. A system for detecting of infants with pain from normal infants based on multi-band spectral entropy by infant's cry analysis. In: Second International Conference on Computer and Electrical Engineering. Dubai: IEEE (2009) p. 72–6. 10.1109/ICCEE.2009.164

[5] Peralta-Malváez L López-Rincón O Rojas-Velazquez D Valencia-Rosado LO Rosas-Romero R Etcheverry G. Newborn cry nonlinear features extraction and classification. J Intell Fuzzy Syst. (2018) 34:3281–9. 10.3233/JIFS-169510

[6] Bano S RaviKumar KM. Decoding baby talk: a novel approach for normal infant cry signal classification. In: International Conference on Soft-Computing and Networks Security (ICSNS). Taipei: IEEE (2015) p. 1–4. 10.1109/ICSNS.2015.7292392

[7] Sailor HB Patil HA. Auditory filterbank learning using ConvRBM for infant cry classification. In: INTERSPEECH. (2018) p. 706–10. 10.21437/Interspeech.2018-1536

[8] Anders F Hlawitschka M Fuchs M. Automatic classification of infant vocalization sequences with convolutional neural networks. Speech Commun. (2020) 119:36–45. 10.1016/j.specom.2020.03.003

[9] Lim WJ Muthusamy H Vijean V Yazid H Nadarajaw T Yaacob S. Dual-tree complex wavelet packet transform and feature selection techniques for infant cry classification. J Telecommun Electron Comput Eng. (2018) 10:75–9. Available online at: https://jtec.utem.edu.my/jtec/article/view/4098

[10] Ji C Xiao X Basodi S Pan Y. Deep learning for asphyxiated infant cry classification based on acoustic features and weighted prosodic features. In: International Conference on Internet of Things (iThings) and IEEE Green Computing and Communications (GreenCom) and IEEE Cyber, Physical and Social Computing (CPSCom) and IEEE Smart Data (SmartData). IEEE (2019) p. 1233–40. 10.1109/iThings/GreenCom/CPSCom/SmartData.2019.00206

[11] Dewi SP Prasasti AL Irawan B. The study of baby crying analysis using MFCC and LFCC in different classification methods. In: IEEE International Conference on Signals and Systems (ICSigSys). Bandung: IEEE. (2019) p. 18–23. 10.1109/ICSIGSYS.2019.8811070

[12] Felipe GZ Aguiar RL Costa YM Silla CN Brahnam S Nanni L. Identification of infants' cry motivation using spectrograms. In: International Conference on Systems, Signals and Image Processing (IWSSIP). Osijek: IEEE. (2019) 181–6. 10.1109/IWSSIP.2019.8787318

[13] Gujral A Feng K Mandhyan G Snehil N Chaspari T. Leveraging transfer learning techniques for classifying infant vocalizations. In: IEEE EMBS International Conference on Biomedical and Health Informatics (BHI), IEEE. (2019) p. 1–4. 10.1109/BHI.2019.8834666

[14] Yuan Y He Q Luo X Shang M. A Multilayered-and-Randomized Latent Factor Model for High-Dimensional and Sparse Matrices. IEEE TransactBig Data. (2020). 10.1109/TBDATA.2020.2988778. [Epub ahead of print].

[15] Yuan Y Luo X Shang MS. Effects of preprocessing and training biases in latent factor models for recommender systems. Neurocomputing. (2018) 275:2019–30. 10.1016/j.neucom.2017.10.040

[16] Yuan Y Luo X Shang M Wu D. A generalized and fast-converging non-negative latent factor model for predicting user preferences in recommender systems. In: Proceedings of The Web Conference. (2020) p. 498–507. 10.1145/3366423.3380133

[17] Luo X Yuan Y Zhou M Liu Z Shang M. Non-negative latent factor model based on β-divergence for recommender systems. IEEE Transact Syst. (2019). 10.1109/TSMC.2019.2931468. [Epub ahead of print].

[18] Yuan Y Xun G Jia K Zhang A. A multi-view deep learning method for epileptic seizure detection using short-time fourier transform. In: Proceedings of the 8th ACM International Conference on Bioinformatics, Computational Biology, Health Informatics. Boston (2017) p. 213–22. 10.1145/3107411.3107419

[19] Ouelha S Touati S Boashash B. An efficient inverse short-time Fourier transform algorithm for improved signal reconstruction by time-frequency synthesis: optimality and computational issues. Digit Sign Proc.(2017) 65:81–93. 10.1016/j.dsp.2017.03.002

[20] Decorsière R Søndergaard PL MacDonald EN Dau T. Inversion of auditory spectrograms, traditional spectrograms, and other envelope representations. IEEE/ACM Transact Audio Speech Lang Proc. (2014) 23:46–56. 10.1109/TASLP.2014.2367821

[21] Flandrin P. Time–frequency filtering based on spectrogram zeros. IEEE Sign Proc Lett. (2015) 22:2137–41. 10.1109/LSP.2015.2463093

[22] Sanchez-Riera J Srinivasan K Hua KL Cheng WH Hossain MA Alhamid MF. Robust RGB-D hand tracking using deep learning priors. IEEE Transact Circ Syst Video Technol. (2017) 28:2289–301. 10.1109/TCSVT.2017.2718622

[23] Yamashita R Nishio M Do RKG Togashi K. Convolutional neural networks: an overview and application in radiology. Insights Into Imaging. (2018) 9:611–29. 10.1007/s13244-018-0639-9 29934920 PMC6108980

[24] Tu F Yin S Ouyang P Tang S Liu L Wei S. Deep convolutional neural network architecture with reconfigurable computation patterns. IEEE Transact Very Large Scale Integr Syst. (2017) 25:2220–33. 10.1109/TVLSI.2017.2688340

[25] Scholkopf B Smola AJ. Learning With Kernels: Support Vector Machines, Regularization, Optimization, and Beyond. Adaptive Computation and Machine Learning Series. Vienna: IEEE (2018). 10.7551/mitpress/4175.001.0001

[26] Alam S Kang M Pyun JY Kwon GR. Performance of classification based on PCA, linear SVM, and Multi-Kernel SVM. In: 2016 Eighth International Conference on Ubiquitous and Future Networks (ICUFN). IEEE (2016) p. 987–9. 10.1109/ICUFN.2016.7536945

[27] Chang CY Chang CW Kathiravan S Lin C Chen ST. DAG-SVM based infant cry classification system using sequential forward floating feature selection. Multidimension Syst Sign Proc. (2017) 28:961–76. 10.1007/s11045-016-0404-5

[28] Chen ST Srinivasan K Lin C Chang CY. Neonatal cry analysis and categorization system via directed acyclic graph support vector machine. In: Big Data Analytics for Sensor-Network Collected Intelligence. (2017) p. 205–22. 10.1016/B978-0-12-809393-1.00010-6

[29] Ashwini K Durai Raj Vincent PM. A deep convolutional neural network based approach for effective neonatal cry classification. Recent Adv Comput Sci Commun. (2020). 10.2174/2666255813999200710135408. [Epub ahead of print].

